# The Freestall Reimagined: Effects on Stall Hygiene and Space Usage in Dairy Cattle

**DOI:** 10.3390/ani11061711

**Published:** 2021-06-08

**Authors:** Annabelle Beaver, Emma Strazhnik, Marina A. G. von Keyserlingk, Daniel M. Weary

**Affiliations:** 1 Animal Welfare Program, Faculty of Land and Food Systems, University of British Columbia, 2357 Main Mall, Vancouver, BC V6T 1Z4, Canada; abeaver@harper-adams.ac.uk (A.B.); emma.straz@gmail.com (E.S.); nina@mail.ubc.ca (M.A.G.v.K.); 2 Department of Veterinary Health and Animal Sciences, Harper Adams University, Newport, Shropshire TF10 8NB, UK

**Keywords:** resting behaviour, standing behaviour, free stall, cubicle, restrictive housing, cow cleanliness, cow comfort, animal welfare, animal well-being

## Abstract

**Simple Summary:**

Lying stalls for dairy cattle are designed to maintain cow hygiene, reduce labor associated with bedding maintenance, and provide cows with a comfortable place to lie down. These considerations can conflict: stall features that, e.g., reduce manure contamination of bedding can make the stall less comfortable, explaining why cows prefer lying in more open spaces. We developed an “alternative” lying area in which traditional freestalls (i.e., in which cattle are not confined to stalls but can move “freely” about the pen) were modified to create larger areas, and flexible stall partitions were included to help maintain cleanliness. We assessed cattle lying behaviour, including lying postures, in this alternative pen compared to both traditional freestalls and an open pack. Not surprisingly, cleanliness was higher in freestalls, but the alternative pen offered substantial improvement in cleanliness over the open pack. There was little difference in postures associated with lying positions (such as lying with limbs outstretched) between the open pack and alternative pen, and both offered greater limb extension compared to freestalls. We conclude that this type of alternative pen can provide producers with the opportunity to improve comfort compared to freestall housing and improve cleanliness compared to housing in an open pack.

**Abstract:**

Modern freestall barns for dairy cattle have been constructed with considerations for dairy cow cleanliness; partitions and other stall features such as neck rails are designed to reduce manure contamination of bedding and decrease farm labor. However, cows prefer to lie in more open spaces, including on bedded packs and pasture. We created an “alternative” housing area by modifying a traditional freestall pen and including flexible partitions to create larger lying areas. We assessed cattle lying behaviour, including lying postures, in this alternative pen (ALT) compared to an open pack (OP) and freestalls (FS) with different stocking densities. We also assessed levels of manure contamination across systems. Cleanliness was highest in FS, but ALT provided substantial improvement compared to OP. Cattle spent more time lying down in OP and ALT compared to FS. There were few differences in postures (such as lying with limbs outstretched) between OP and ALT, but cows in both of these systems more often lay in extended positions compared to when they were housed in FS. Housing in OP and ALT was associated with reduced perching for cows with high body weight; perching has been linked to an increased prevalence of both hoof lesions and lameness. Thus, alternative lying areas can offer a solution for producers seeking to provide cattle with the advantages of a more open lying area, while improving hygiene relative to an open pack.

## 1. Introduction

Modern indoor housing systems for dairy cattle are designed to help keep the animals and facility clean with minimal labour. Stall partitions and other design features of freestalls modify cow behaviour to discourage defecation onto the bedding surface (see [1]). Reducing the occurrence of this behaviour is a priority, since soiled bedding is linked to contaminated udders and an increased risk of mastitis [2,3,4]. Previous studies have shown that modified stall designs, including wider freestalls [5] and neck rails moved further from the curb [6,7], can increase the risk that cows defecate onto the bedded area. However, these same design elements improve stall usage by dairy cows and decrease the risk of lameness [8,9]; thus, the difficulty in optimizing cleanliness while reducing lameness prevalence has created a type of paradox in good stall design [9].

When provided a choice between open packs and freestalls, dairy cattle show a strong preference for more open spaces for both standing and lying [10]. Dairy cows spend less time lying in freestalls compared to pasture [11], “open pack” sand packs [10], and more open stalls with alternative or flexible partitions [12]. Cows also prioritize space above other aspects of housing such as bedding material [13]. Open lying areas are thought to allow for a wider range of lying postures. For example, cows spend more time with their head resting backwards on their body or outstretched on the ground when on pasture compared to in tiestalls [14]; these results are of special interest given the association between head position and rapid eye-movement (REM) sleep [15]. Little work to date has investigated the effect of freestall design on body position during rest.

The primary aim of the current study was to investigate differences in lying behaviour, including lying postures, in dairy cattle tested in different housing systems (specifically an open pack, a common freestall design, and an alternative freestall designed to provide a less restrictive lying area). Given the consequences of stall design on fecal soiling of the lying surface, a secondary aim was to quantify the effect of these different housing systems on bedding cleanliness. As the amount of space available per cow may affect both outcomes, and treatments differed in space available, we tested the freestall treatment at two stocking levels, fully stocked and stocked at 50% (i.e., one cow per two stalls).

## 2. Materials and Methods

### 2.1. Experimental Setup and Management

**Figure 1:**
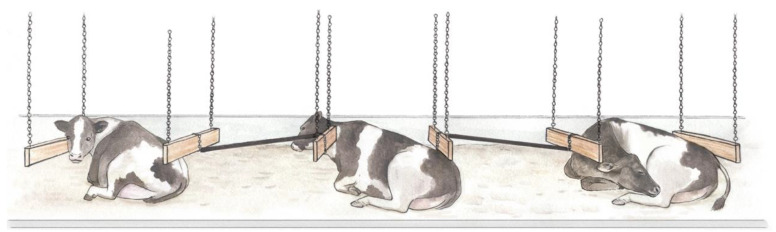
Illustration of the alternative stalls. Two rows, each of six traditional freestalls, were modified to create three large “stalls” (each 2.4 m wide; only one row is illustrated here). The hanging partitions delineated the lying area, and ABS plastic piping prevented cows from entering the area between adjacent stalls. Drawing by Ann Sanderson.

Animals were housed at The University of British Columbia’s Dairy Education and Research Centre in Agassiz, BC, Canada. In total, 48 non-lame, dry, pregnant Holstein cows (mean ± SD parity = 2.1 ± 1.5; BW = 740 ± 100 kg) were included in the study. Cows were fed a total mixed ration (TMR) once per day, consisting of 30.1% straw, 26.75% grass silage, 21.86% alfalfa hay, 20.6% corn silage, and 0.7% dry cow mineral composition. The animals were fed at 08:30, and feed was pushed up three times per day. Water was provided through one self-filling trough in each pen, cleaned once weekly. Cows were enrolled 54 ± 9 d before expected calving date and blocked into eight “groups” of six based on dry-off date. Individuals in each group were dried off together and moved to their respective experimental pen. To facilitate individual identification, animals were marked with unique symbols using hair dye two days prior to their move to the experimental setup. The treatments were as follows:

1. Freestall, 50% stocking (FS1): Animals were housed in a conventional freestall pen fitted with two rows of six stalls (see [16] for a schematic diagram). This pen was stocked with six cows (i.e., one “group”), corresponding to a 50% stocking density. Each stall was 1.2 m wide and deep-bedded with 0.4 m of washed river sand, replenished weekly. The neck rail was 1.7 m from the rear curb (measured horizontally) and approximately 1.2 m above the bedding. The rear curb was 18 cm high, and there was a concrete brisket 2 m from the inside edge of the rear curb. Sand was leveled daily with a rake between brisket and curb. The flooring, including crossovers between alleys, was textured rubber matting. The alleys between stall banks, and in front of the feed bunk, were cleaned four times per day using a cable-driven scraping system (GEA Houle Inc., Drummondville, QC, Canada). The feed bunk was accessible via 12 evenly spaced headlocks.
2. Freestall, 100% stocking (FS2): Housing and management for this treatment were as described for FS1, but the pen was stocked at 100% (i.e., with 12 cows). This stocking density was achieved by housing two “groups” of six animals in the same pen.
3. Open Pack (OP): All stall partitions and the neck rail were removed, with the exception of the partitions separating each row of stalls within the pen from the crossovers, leaving two “open pack” areas of 15.2 m^2^ each. The pen was stocked with six cows (i.e., one “group”). Feeding and management were as previously described.
4. Alternative Stall (ALT): Each row of six stalls was modified to create three large “stalls”, each 2.4 m wide (Figure 1). Modifications consisted of hanging two 120 cm × 5 cm × 20 cm wooden boards perpendicular to the rear curb using chains from the ceiling. The two boards were spaced at 2.4 m and suspended 0.5 m above the stall. The space between each of the stalls was blocked using ABS (acrylonitrile butadiene styrene) plastic piping attached by a chain to adjacent hanging boards. The total lying area in the ALT pen (including the area under the piping) was 30.4 m^2^. This pen was stocked with 6 cows (i.e., one “group”).

Each of the eight groups was tested for a seven-day period under each treatment condition. The order in which the treatments were presented to each group was randomized, with the constraint that an equal number of groups began and ended with the control (FS2) or an alternative housing area (OP or ALT). The order of treatments across the six groups is shown in Table S1. Cows were not physically moved between treatments (except for one of two “groups” when combined in FS2); instead, the pen was physically reconfigured during feeding (between 08:00 and 10:00) every seven days. During the first five days of each treatment, cows were allowed to habituate to the pen and behavioural data were recorded during the last two days of the treatment period (i.e., Days 6 and 7).

### 2.2. Data Collection

#### 2.2.1. Behavioural Observations

Three cameras were mounted over the experimental pen: two dome cameras (model WV-CW504, Panasonic, Kadomashi, Japan) were mounted over the feed alley and the lying area closest to the feed bunk. One camera (model WV-CP310, Panasonic, Kadomashi, Japan) was mounted over the rear alley approximately 2.4 m from the window. Video was recorded continuously, and a red lamp (100 W, <5 lux) was used to facilitate nighttime video recording. 

We developed an ethogram (Table 1) to measure standing and lying postures. Behaviours and postures were initially derived from the ethogram presented by Haley et al. [17]. Cattle were scored using 5 min scans, which have been shown to provide an accurate representation of total lying time [18]. All scans in which a human was visible in the pen were excluded from analysis.

#### 2.2.2. Stall Cleanliness Scoring

Stall cleanliness was calculated twice daily for the last two days of each treatment period. A 1 m × 1.6 m grid, comprised of 160 10 cm × 10 cm squares (as described in [19]), was placed at the outside edge of the curb and centred between the partitions (in FS1, FS2, and ALT) or sequentially placed along the sand bank (in OP) seven times, such that all available lying area was measured. Where manure was deposited at the front end of the freestall (e.g., more than 1.6 m from the rear curb), a mark was made in the sand at the front edge of the grid. The grid was then moved forward so that the edge closest to the curb was now in line with the mark, and the grid now covered the front of the stall. The number of dirty squares counted in both grid placements was summed to represent the total lying area per stall.

The total number of soiled squares from each flooring area was summed across the two observations within a day and then averaged across the two observational days to provide an aggregate measure of lying surface cleanliness. Initially, the area underneath the plastic piping in ALT was not included in cleanliness scoring. However, during testing, we observed that cows occasionally extended limbs underneath this piping, so this flooring area was included beginning with the fourth group of cows; for this reason, data from Groups 1–3 in ALT were excluded from the analysis of stall cleanliness. 

### 2.3. Statistical Analysis

#### 2.3.1. Interobserver Agreement

These analyses were performed using SPSS (IBM, version 26, Armonk, NY, USA) and GraphPad Prism (version 8, GraphPad, San Diego, CA, USA). Absolute agreement between observers for cleanliness scoring was assessed using the interclass correlation coefficient (ICC; two-way random effects model). We calculated separate ICC values for the bedded pack (based on a total of 42 observations) and for the freestalls (based on a total of 72 observations) due to slightly different processes used in obtaining the cleanliness measurements in each of these areas. Three overall Cohen’s kappa coefficients were calculated for behaviour (lying or standing), posture, and head position (as represented in Table 1) when each animal was in view of both observers. Interobserver reliability in head position was calculated from observations in which both observers agreed that the cow was lying down. Kappas were also assessed separately for each environment (ALT, OP, FS1, and FS2) to account for any differences in observation with stocking density and pen structure.

#### 2.3.2. Development of Behavioural Models

All further statistical analyses were performed in SAS (version 9.4, SAS Institute, Cary, NC, USA) and figures were generated using GraphPad Prism (Version 8, San Diego, CA, USA). Data were first studied graphically and descriptively using PROC Univariate and PROC Freq to gain familiarity with the frequencies and distributions of each variable.

We created 2 main behavioural models, with Average Time Spent Perching (per 24 h) and Average Time Spent Standing (per 24 h) each serving as outcomes and Treatment as the predictor of interest. In addition, to explore time spent lying down, we created 4 models to evaluate whether Treatment could predict the amount of time that cows spent lying in certain positions (per total lying time): Lying down with no limbs extended (corresponding to “None” in the ethogram), Lying down with both hind limbs extended (“H2” in the ethogram), Lying down with the head curled (“Curled” in the ethogram), and Lying down stretched out. This final category represented a combination of several lying behaviours recorded in the ethogram; specifically, a cow was recorded as Lying down stretched out if she was either in a full lateral position (“Full” in the ethogram) or if she had both hind legs extended (H2 in the ethogram) in addition to resting her head on its side with neck outstretched (“Down” in the ethogram). These behaviours were combined because “Full” was rare, and the simultaneous occurrence of “H2 + Down” corresponded to a similar space allocation to “Full”.

We generated a correlation matrix of all potential predictor variables to evaluate the assumption of multicollinearity using r ≥ |0.70| as a threshold. Body Weight (BW) and Body Condition Score (BCS) were highly correlated (r = 0.77) and were thus not included simultaneously in any model. Multivariable mixed linear models for the following outcome variables were constructed using PROC Mixed in SAS: Average Time Spent Perching, Average Time Spent Standing, Lying down with no limbs extended (“None”), Lying down with both hind limbs extended (“H2”), and Lying down with the head curled (“Curled”). The variables Treatment (ALT, OP, FS1, and FS2), and Parity (categorized as 1st or ≥2nd), were included as fixed effects in all models regardless of significance level. Other potential fixed-effect predictors were BCS, BW, DCC, and all relevant two-way interactions were retained in the model if significant, using a manual backwards stepwise elimination procedure. Cow ID nested within Group was included as a random effect in all models. Planned contrasts were conducted using the Contrast statement to compare: (1) alternative housing types (ALT and OP) to freestall housing (FS1 and FS2); (2) half versus fully stocked pens (FS1 versus FS2); and (3) open to semi-open housing (OP versus ALT). If an interaction was present between Treatment and a continuous variable, the variable was retained as continuous in the model but was categorized for further exploration with planned contrasts.

Due to low counts in the outcome variable Lying down stretched out, a mixed Poisson regression model for over-dispersed data was developed using PROC GLIMMIX in SAS. Again, Treatment and Parity were included as fixed effects and Cow ID nested within Group as a random effect. Planned contrasts were conducted as previously described.

#### 2.3.3. Development of the Cleanliness Model

By means of PROC Mixed, a mixed linear model was created to evaluate the cleanliness of each housing type. The outcome variable was the proportion of available flooring area contaminated by manure per cow (based on the number of cows present in the pen at a given time). Treatment was considered as the fixed effect of interest, and Group was included as a random effect. Data were averaged across two days of observation, with the exception of one case in ALT in which only one day of data was available. Additionally, due to a data entry error, Week 6 in OP could not be included in the analysis.

Denominator degrees of freedom were calculated using the containment method. Because two different groups were present at one time in the FS2 treatment, and hence were not independent, a manual adjustment was made to the denominator degrees of freedom to reflect an accurate number of unique observations. Of a total of 28 observations, only 24 were unique; thus, the denominator degrees of freedom were reduced from 17 to 13 using the “ddf” = option in SAS. Planned contrasts were conducted using the contrast statement.

#### 2.3.4. Model Fit

One of the outcome variables (Average Time Spent Perching) required a cube-root transformation to achieve normality of model residuals. We selected models with the lowest Akaike information criterion (with correction for small sample sizes), with the stipulation that the variables Treatment and Parity were always retained. A variance components (VC) covariance structure was selected for all models, as no model improvements were identified when the structure was altered. For linear models in which the outcome was a proportion, all predicted values were in a plausible range (i.e., between 0 and 1).

## 3. Results

### 3.1. Interobserver Agreement 

The ICCs (95% confidence intervals, CI) for cleanliness scoring were 0.98 (0.97, 0.99) in the bedded pack and 0.99 (0.99, 1.0) in the freestalls. Although only two raters were used, the lower bound of the 95% CI for both ICCs was above 0.90, indicating excellent agreement [20]. 

A total of 13,825 observations from each of the two observers was available for assessing interobserver reliability in behaviour, posture, and head position. The observations spanned 8 days and included observations from 24 animals. There was near-perfect agreement for behaviour (kappa (95% CI) = 0.99 (0.99, 1.0) according to criteria outlined by Landis and Koch [21], and the environment-specific kappa ranged from 0.99 (in OP) to 1.0 (in FS2). There was also near-perfect agreement for head position (kappa (95% CI) = 0.94 (0.92, 0.95), with the environment-specific kappa ranging from 0.91 (in FS2) to 0.97 (in ALT). For posture, the overall agreement was substantial [21] (kappa (95% CI) = 0.76 (0.75, 0.77), with the environment-specific kappa ranging from 0.70 (FS1; substantial agreement) to 0.81 (ALT; near-perfect agreement).

### 3.2. Standing and Perching Behaviour

The proportion of time spent standing (per 24 h) did not differ between treatments, with cows spending an average (±SE) of 43% (±0.9%) of their time standing; however, planned contrasts revealed increased lying time (*p* = 0.02) in alternative housing systems (i.e., OP and ALT) compared to freestalls (i.e., FS1 and FS2). BCS was a predictor of standing time, such that standing decreased as BCS increased (F_1,280_ = 6.03, *p* = 0.015) (for full model results, see Table S2).

**Figure 2:**
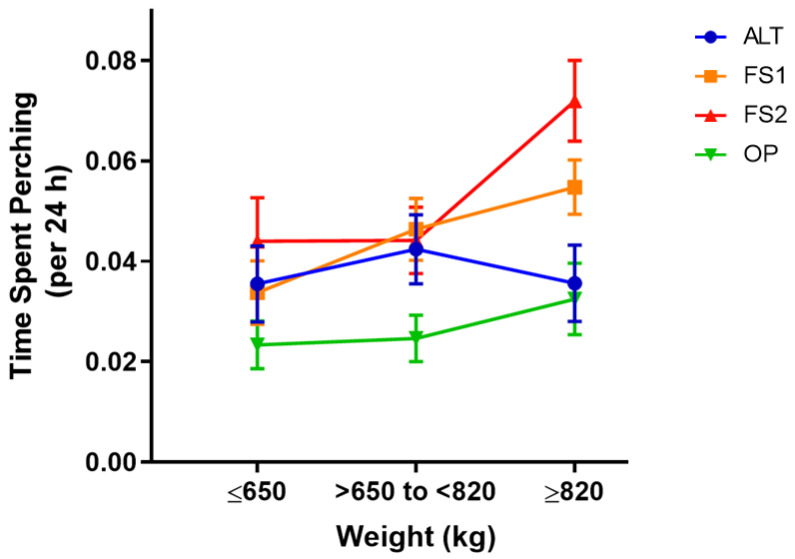
Interaction plot depicting the relationship between weight (categorized into three groups for ease of interpretation) and treatment on the average proportion of time spent perching (per 24 h). Weight categories are shown on the x-axis, with ≤650, >650 to <820, and ≥820 kg, representing the 25th, 50th, and 75th weight percentiles, respectively. The proportion of time spent perching is shown on the y-axis. Different colored lines represent the treatments: Alternative (ALT), Half-Stocked Freestalls (FS1), Fully Stocked Freestalls (FS2), and Open Pack (OP). Error bars represent ±1 SEM.

There was an interaction between treatment and BW on Perching (F_3,270_ = 6.53, *p* < 0.001; Table S3). Planned contrasts indicated that, for high BW cows (>820 kg, the 75th percentile), perching was reduced in alternative housing areas (OP and ALT) compared to freestalls (FS1 and FS2) (*p* < 0.001), in half versus fully stocked freestalls (FS1 versus FS2) (*p* < 0.001), and in OP compared to ALT (*p* = 0.019). For low BW cows (≤650 kg, the 25th percentile), the only reduction in time spent perching was between OP and ALT (*p* = 0.002) (Figure 2).

### 3.3. Body Postures While Lying Down

**Figure 3:**
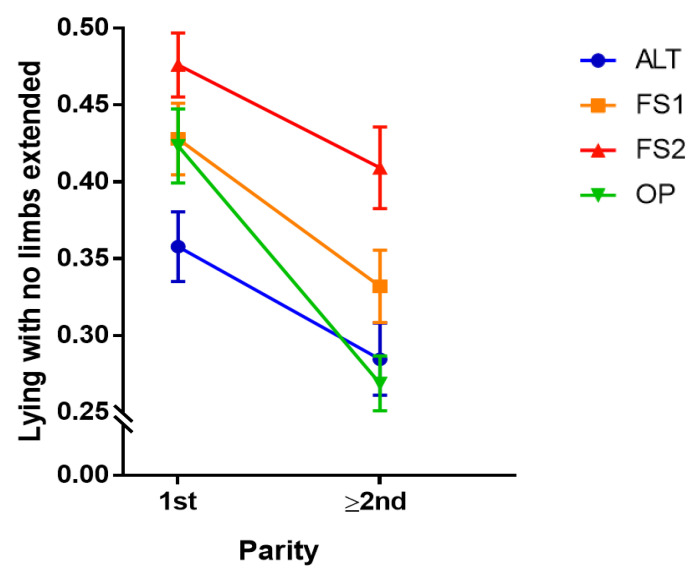
Interaction plot depicting the relationship between parity (categorized into 1st and ≥2nd parity) and treatment on the time spent lying down with none of the four limbs extended (per total lying time). Parity categories (1st and ≥2nd) are shown on the x-axis. The proportion of time spent lying down with no limbs extended is shown on the y-axis. Different colored lines represent different treatments: Alternative (ALT), Half-Stocked Freestalls (FS1), Fully Stocked Freestalls (FS2), and Open Pack (OP). Error bars represent ±1 SEM.

There was an interaction between treatment and parity on the amount of time cows spent lying down with no limbs extended (F_3,277_ = 2.79, *p* = 0.041, Figure 3 and Table S4), with 1st parity cows spending more in this posture in ALT compared to OP (*p* < 0.001). This difference was no longer evident for cows in higher parities. Both 1st and ≥2nd parity cows spent more time in this posture in freestalls (FS1 and FS2) compared to alternative housing areas (ALT and OP) and in half versus fully stocked pens (FS1 versus FS2) (*p* < 0.001).

**Figure 4:**
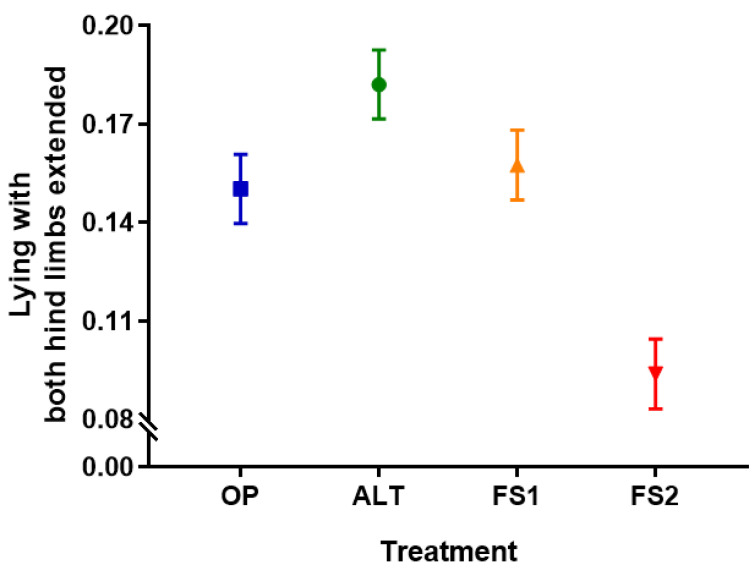
Least squared mean (±SE) proportion of time spent lying down with both hind limbs stretched for each of the four treatments: Alternative (ALT), Half-Stocked Freestalls (FS1), Fully Stocked Freestalls (FS2), and Open Pack (OP).

Cows in parity 2 or higher spent more time lying with both hind limbs extended (F_1,318_ = 7.75, *p* = 0.006), as did cows with higher BCS (F_1,318_ = 4.40, *p* = 0.037). There was also an effect of treatment (F_3,318_ = 12.40, *p* < 0.001, Figure 4 and Table S5): cows spent more time in this position in alternative housing systems (ALT and OP) compared to freestalls (FS1 and FS2) (*p* < 0.001), in half versus fully stocked freestalls (FS1 versus FS2) (*p* < 0.001), and in ALT compared to OP (*p* = 0.033).

**Figure 5:**
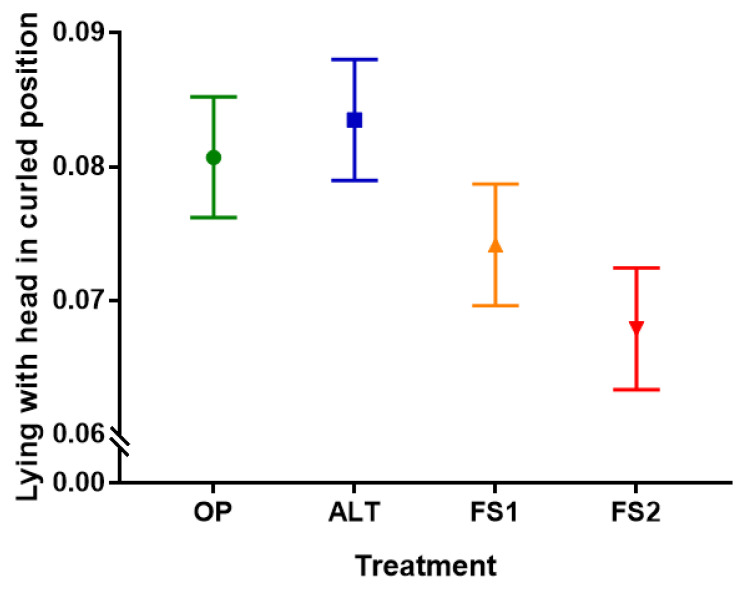
Least squared mean (±SE) proportion of time spent lying down with the head curled for each of the four treatments: Alternative (ALT), Half-Stocked Freestalls (FS1), Fully Stocked Freestalls (FS2), and Open Pack (OP).

First lactation cows spent more time lying with their heads in a curled position (F_1,280_ = 8.44, *p* = 0.001). There was also an effect of treatment on time spent in this posture (F_3,280_ = 5.96, *p* = 0.004, Table S6): cows spent more time in this position in alternative housing systems (ALT and OP) compared to freestalls (FS1 and FS2) (*p* < 0.001) and in half versus fully stocked freestalls (FS1 versus FS2) (*p* = 0.001). There was no difference between the ALT and OP treatments (Figure 5).

The number of times that cows were observed lying down in a stretched-out position also differed between treatments (F_3,280_ = 5.98, *p* = 0.001, Table S7). Cattle were more frequently observed in this position in open housing treatments (ALT and OP) compared to freestalls (FS1 and FS2) (*p* < 0.001) and in half versus fully stocked pens (FS1 versus FS2) (*p* = 0.014). There was no difference between ALT and OP treatments.

### 3.4. Cleanliness

**Figure 6:**
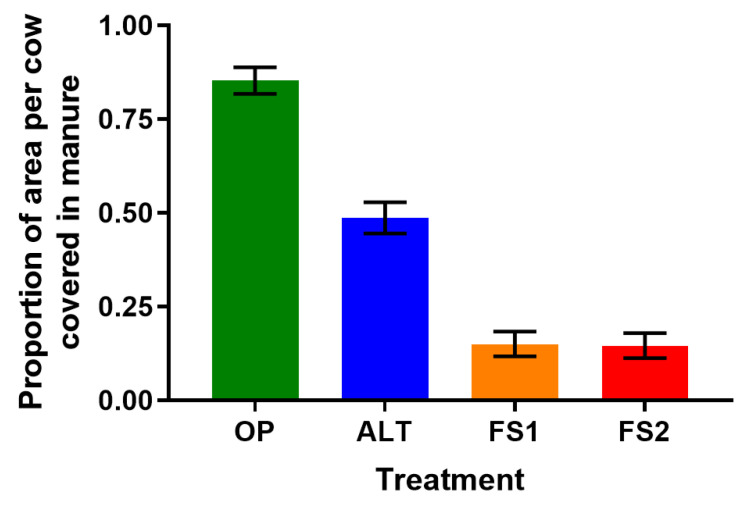
Least squared mean (±SE) proportion of flooring area with visible manure for each of the four treatments: Alternative (ALT), Half-Stocked Freestalls (FS1), Fully Stocked Freestalls (FS2), and Open Pack (OP).

The percentage (±SE) of flooring area per cow contaminated with manure varied with treatment (*p* < 0.001, Figure 6 and Table S8). Planned contrasts showed improved cleanliness (*p* < 0.001) in freestalls (FS1 and FS2) versus alternative housing areas (i.e., OP and ALT), and in semi-open versus open housing areas (i.e., ALT versus OP).

## 4. Discussion

Previous studies on cattle housing have largely focused on time budgets for standing and lying; these studies have demonstrated reduced stall usage when the space available is more restrictive [5,6,7]. In the present study, we also found that cows spent more time lying in alternative housing systems (both OP and ALT) compared to freestalls, regardless of stocking density, which corresponds well with earlier work. It is worth noting that lying time was reduced in freestalls despite the compliance of our freestall width (1.2 m) with industry recommendations in Canada [22]. 

The majority of dairy cattle in North America are housed indoors [23], so research on indoor housing options is of continued importance. Overall, we found no differences in lying time between cattle in ALT and OP. An absence of evidence does not equate to an evidence of absence, but the results are encouraging, as lying behaviour is highly motivated in dairy cattle [24,25] and represents a large proportion of their time budget [26].

Perching, in which cows stand with two feet in the stall and the other feet in the alley, is commonly observed in freestalls. A high proportion of time perching is associated with difficulties in stall usage resulting from poorly placed stall hardware including neck rails and stall partitions [5,27]. Importantly, perching has been associated with an increased risk of lameness [28] and a higher number of soft-tissue lesions of the hoof [29]. Roughly 65% of dairy cows in Canada are unable to “fit” into the average freestall [30], explaining the interaction between cow BW and perching in the current study. Exposure to alternative housing areas (both OP and ALT) reduced perching for cows with high BW (>820 kg) when compared to traditional freestall housing. This was not the case for smaller animals, (BW ≤ 650 kg), presumably because they had less difficulty navigating the hardware in the freestall.

An important feature of the current study is that we also examined the effect of treatments on lying postures. Some previous research has found associations with head posture and housing, with cows spending more time lying with their heads back or resting on the ground when kept on pasture compared to in tiestalls [31]. In the current study cows used extended lying positions more frequently in alternative systems (OP and ALT) compared to freestalls. Specifically, cattle spent more time with both hind legs extended, with the neck curled back, and in a full lateral position in the alternative systems compared to freestalls. Importantly, no compromises in lying postures were observed when comparing ALT to OP, including time spent stretched out, and with the neck curled back. Indeed, more cows in ALT vs. OP were observed to lie with both hind limbs extended. Given cattle prefer to lie down in open areas [10,11], we hypothesize that these body postures are indicative of improved comfort. Although difficult to define, “cow comfort” is of great interest to both the industry [32] and animal welfare organizations [33]. A limitation of our work is that it was conducted with dry cows; further studies are necessary to make inferences about the effects of these modified housing systems for lactating cattle, as well as during different stages of lactation. Additionally, future studies could examine the association of specific lying postures with sleep quality to make stronger inferences about comfort in these housing environments. There is currently little research regarding the interaction between quality of sleep and lying positions, beyond that the head is not held upright during REM sleep [15,34].

We found an interaction between treatment and parity on the time cows spent lying down with no limbs extended. Cows of first parity and higher assumed this posture more frequently in freestalls compared to alternative lying areas (OP and ALT), and in half versus fully stocked pens. However, first parity cows assumed this position more often in ALT compared to OP. This finding was somewhat surprising, as we might expect less behavioural plasticity in older cows habituated to freestalls. The current results suggest that factors associated with age, such as larger udders or increased BW, may make these postures especially important.

Not surprisingly, cleanliness level was highest in freestalls: hygiene is a main factor influencing the design of these systems [1]. More interestingly, we noted improved cleanliness in ALT compared to OP. Thus, the ALT design can be seen as a type of compromise in addressing the negative relationship between “cow comfort” and stall cleanliness [9]. Examples of other efforts to address this issue includes the “High Welfare Floor” [35], which is layered with textile, foam, a void-forming storage unit, and an impermeable membrane; this flooring can provide an open lying area and maintain cleanliness via separation of urine from feces. An advantage of the ALT system in the present study is that it can be constructed by modifying existing freestalls. We encourage future work to examine other creative alternative systems that seek to better balance comfort and cleanliness, with the additional empirical measurements of udder hygiene and hoof health. According to the authors of [36], innovative combinations of cubicle and Freewalk housing (i.e., loose housing without cubicles), and multifunctional buildings that accommodate multiple systems, show promise for the future of dairy cattle housing. 

## 5. Conclusions

This study documented the effects of several housing methods on lying time and specific lying postures associated with cow comfort. We found that postures such as lying with outstretched limbs, or with the neck curled, were least frequently observed in freestalls. Importantly, these postures were not compromised in the alternative housing pen compared to the open pack. Although stall cleanliness was highest in freestalls, the alternative system offered improvements in cleanliness compared to the open pack. Thus, we conclude that a modified lying area, such as that provided by the alternative treatment, can offer improved cleanliness in comparison with an open pack, and improved cow comfort relative to freestall housing. 

## Figures and Tables

**Table 1:** Ethogram detailing the standing and lying behaviours scored from video recordings.

POSTURE	DESCRIPTION
**Standing**	Any position in which any limb bears weight (including the process of standing up and lying down)
Alley	>3 feet placed in any alley or within a crossover; includes feeding
Perching	1 or 2 feet (but no more) placed within the stall (either on the bedding or rear curb)
**Lying**	Any position with full weight resting on sternum or flank
*Leg positions* None	Sternal recumbency with no limbs extended; hock joint close to body, no space or visible bedding between body and metatarsus.
H1	Sternal recumbency with hind leg visible. Hock joint either touching, or positioned away from, the body. Bedding visible in an angle up to 45° between the body and metatarsus.
H2	Sternal recumbency with both hind legs visible. Top leg creates a >45° angle with body. At least part of bottom hind leg visible between body and top leg.
Full	Lateral recumbency. At least one front and one hind leg extends from the body at a >45° angle.
*Head position* Up	Head not resting on any part of body, bedding, or structure
Down	Head resting on its side (either on bedding, legs, or other structure), with neck outstretched
Curled	Neck curled back, head resting on body

## Data Availability

The data used in this study are available from the corresponding author upon reasonable request.

## Supplementary Materials

The following are available online at https://www.mdpi.com/article/10.3390/ani11061711/s1, Table S1: Order of treatments within group, Table S2: Final mixed-effects linear regression model of standing time, Table S3: Final mixed-effects linear regression model of perching time, Table S4: Final mixed-effects linear regression model of lying down with no limbs extended, Table S5: Final mixed-effects linear regression model of time spent lying down with both hind limbs extended, Table S6: Final mixed-effects linear regression model of time spent lying with head in a curled position, Table S7: Final mixed-effects Poisson regression model of lying down stretched out, Table S8: Final mixed-effects linear regression model of cleanliness.
