# Supplementary material for: The Freestall Reimagined: Effects on Stall Hygiene and Space Usage in Dairy Cattle
Source: Animals (Basel). 2021 Jun 8;11(6):1711. doi: 10.3390/ani11061711 (PMC8228901; doi:10.3390/ani11061711)
Supplement: Supplementary file 1 [file animals-11-01711-s001.zip › animals-1212350-SI.pdf]

Supplementary Tables

Table S1. Order of treaments within group. The treatments represented are ALT: Alternative stall; OP: Open pack; FS1: Freestall, 50% stocking; FS2: Freestall, 100% stocking.

		Treatment Order			
**Group**		**1**	**2**	**3**	**4**
	**1**	ALT	OP	FS1	FS2
	**2**	FS2	FS1	OP	ALT
	**3**	ALT	OP	FS1	FS2
	**4**	FS2	FS1	ALT	OP
	**5**	OP	ALT	FS1	FS2
	**6**	FS2	FS1	OP	ALT
	**7**	OP	ALT	FS1	FS2
	**8**	FS2	FS1	ALT	OP

Table S2. Final mixed-effects linear regression model with *Time Spent Standing* (per 24 h) as the outcome and *Cow* nested within *Group* as a random effect. Slope (β) estimates for all included fixed effects are presented in addition to the model intercept. Standard errors of the estimates (SE), degrees of freedom (DF), t values, and associated *P* values are also presented.

**Effect**	**Estimate**	**SE**	**DF**	**t Value**	**P > |t|**
Intercept	0.667	0.100	38	6.67	< 0.0001
Treatment					
ALT	0.001	0.007	280	0.13	0.896
FS1	0.013	0.007	280	1.88	0.062
FS2	0.010	0.007	280	1.51	0.131
OP (ref)	-	-	-	-	-
Parity					
1	-0.021	0.019	280	-1.09	0.275
≥ 2 (ref)	-	-	-	-	-
BCS	-0.067	0.027	280	-2.43	0.016

Table S3. Final mixed-effects linear regression model with the cube-root transformation of *Average Time Spent Perching* (per 24 h) as the outcome and *Cow* nested within *Group* as a random effect. Slope (β) estimates for all included fixed effects are presented in addition to the model intercept. Standard errors of the estimates (SE), degrees of freedom (DF), t values, and associated *P* values are also presented.

**Effect**	**Estimate**	**SE**	**DF**	**t Value**	**P > |t|**
Intercept	0.0760	0.132	37	0.58	0.568
Treatment					
ALT	0.162	0.066	270	2.45	0.015
FS1	-0.050	0.067	270	-0.76	0.451
FS2	-0.074	0.067	270	-1.11	0.267
OP (ref)	-	-	-	-	-
Parity					
1	0.020	0.036	270	0.56	0.577
≥ 2 (ref)	-	-	-	-	-
BW	0.0002	0.0002	270	1.36	0.176
BW*Treatment					

**Effect**	**Estimate**	**SE**	**DF**	**t Value**	**P > |t|**
BW*ALT	-0.0001	0.0001	270	-1.42	0.158
BW*FS1	0.0002	0.0001	270	1.95	0.052
BW*FS2	0.0002	0.0001	270	2.51	0.013
BW*OP (ref)	-	-	-	-	-

Table S4. Final mixed-effects linear regression model with *Proportion of time spent lying down with no limbs extended* (per total lying time) as the outcome and *Cow* nested within *Group* as a random effect. Slope (β) estimates for all included fixed effects are presented in addition to the model intercept. Standard errors of the estimates (SE), degrees of freedom (DF), t values, and associated *P* values are also presented.

Effect	***Estimate***	***SE***	***DF***	***t Value***	***P > |t|***
Intercept	0.269	0.029	39	9.27	<0.0001
Treatment					
ALT	0.016	0.022	277	0.73	0.466
FS1	0.063	0.022	277	2.92	0.004
FS2	0.140	0.022	277	6.48	<0.0001
OP (ref)	-		-	-	-
Parity					
1	0.155	0.042	277	3.72	0.0002
≥ 2 (ref)	-	-	-	-	-
Treatment*Parity (ref= OP, ≥ 2 parity)					
ALT*1st	-0.081	0.031	277	-2.62	0.009
FS1*1st	-0.044	0.031	277	-1.39	0.164
FS2*1st	-0.073	0.031	277	-2.32	0.021

Table S5. Final mixed-effects linear regression model with *Proportion of time spent lying down with both hind limbs extended* (per total lying time) as the outcome and *cow* nested within *group* as a random effect. Slope (β) estimates for all included fixed effects are presented in addition to the model intercept. Standard errors of the estimates (SE), degrees of freedom (DF), t values, and associated *P* values are also presented.

Effect	*Estimate*	*SE*	*DF*	*t Value*	*P > |t|*
Intercept	0.041	0.064	318	0.64	0.523
Treatment					
ALT	0.032	0.015	318	2.14	0.033
FS1	0.007	0.015	318	0.49	0.624
FS2	-0.056	0.015	318	-3.77	0.0002
OP (ref)	-	-	-	-	-
Parity					
1	-0.034	0.012	318	-2.78	0.006
≥2 (ref)	-	-	-	-	-
BCS	0.036	0.017	318	2.10	0.037

Table S6. Final mixed-effects linear regression model with *Proportion of time spent lying down with the head in a curled position* as the outcome and cow nested within group as a random effect. Slope (β) estimates for all included fixed effects are presented in addition to the model intercept. Standard errors of the estimates (SE), degrees of freedom (DF), t values, and associated *P* values are also presented.

Effect	*Estimate*	*SE*	*DF*	*t Value*	*P* > |*t*|
Intercept	0.070	0.006	39	11.94	< 0.0001
Treatment					
ALT	0.003	0.004	280	0.70	0.485
FS1	-0.007	0.004	280	-1.62	0.107
FS2	-0.013	0.004	280	-3.18	0.002
OP (ref)	-	-	-	-	-
Parity					
1st	0.022	0.008	280	2.90	0.004
≥ 2nd (ref)	-	-	-	-	-

Table S7. Final mixed-effects Poisson regression model with *Count of lying down stretched out* as the outcome and *Cow* nested within *Group* as a random effect. Slope (β) estimates for all included fixed effects are presented in addition to the model intercept. Standard errors of the estimates (SE), degrees of freedom (DF), t values, and associated *P* values are also presented.

**Effect**	**Estimate**	**SE**	**DF**	**t Value**	**P > |t|**
Intercept	-0.456	0.247	39	-1.85	0.072
Treatment					
ALT	0.326	0.194	280	1.68	0.094
FS1	-0.342	0.230	280	-1.49	0.138
FS2	-0.535	0.244	280	-2.19	0.029
OP (ref)	-	-	-	-	-
Parity					
1st	-0.314	0.312	280	-1.01	0.315
≥ 2nd (ref)	-	-	-	-	-

Table S8. Final mixed-effects linear regression model with the proportion of pen space covered in manure per cow (*Cleanliness*) as the outcome variable and group as a random effect. Slope (β) estimates for treatment are presented in addition to the model intercept. Standard errors of the estimates (SE), degrees of freedom (DF), t values, and associated *P* values are also presented.

Effect	*Estimate*	*SE*	*DF*	*t Value*	*P* > |*t*|
**Intercept**	0.854	0.035	7	24.18	<0.0001
**Treatment**					
**ALT**	-0.366	0.053	13	-6.87	<0.0001
**FS1**	-0.703	0.047	13	-15.10	<0.0001
**FS2**	-0.707	0.047	13	-15.07	<0.0001
**OP (ref)**	-	-	-	-	-
